# Unexpected community-acquired co-infections during an ongoing pandemic: (one) too many diagnoses at once

**DOI:** 10.1007/s15010-021-01647-0

**Published:** 2021-07-03

**Authors:** David Manuel Cordas dos Santos, Laura Fischer, Michael von Bergwelt-Baildon, Elham Khatamzas

**Affiliations:** 1 Department of Medicine III, LMU University Hospital, Marchioninistraße 15, Munich, 81377 Germany; 2 Cancer- and Immunometabolism Research Group, LMU Gene Center, Munich, Germany; 3 German Cancer Consortium (DKTK), partner site Munich, and German Cancer Research Center (DKFZ), Heidelberg, Germany; 4 COVID-19 Registry of the LMU Munich (CORKUM), LMU University Hospital, Munich, Germany

In November 2020, a 44-year-old man presented to our hospital with a two-week history of flu-like symptoms, night sweats and swollen lymph nodes. The patient stayed in an emergency shelter, reported significant regular alcohol intake (min. 20 units/day) and nicotine abuse (35 pack years). He was febrile, tachypnoeic with no neurological signs. Physical examination was remarkable for generalized peripheral lymphadenopathy and skin lesions (Fig. 1A). Blood film demonstrated leucocytosis with 63% blasts with absolute neutropenia. Histopathological examination of the skin lesions revealed chloroma as an extramedullary manifestation (Fig. 1B/C). Bone marrow examination confirmed the diagnosis of acute monoblastic leucaemia (Fig. 1D/E). Cytoreductive regimen with hydroxyurea was commenced.

**Fig. 1:**
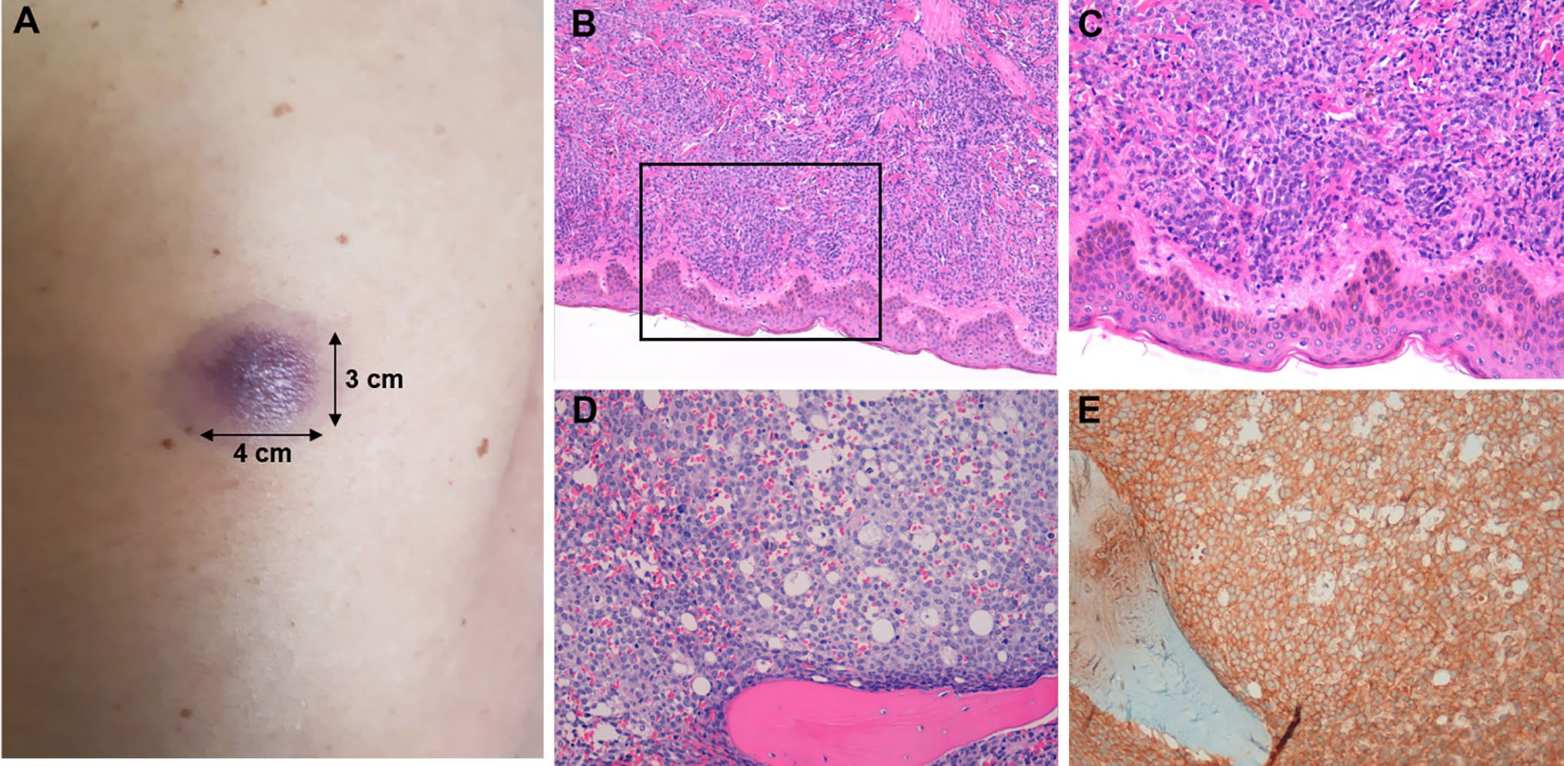
Leukemic blasts infiltrate skin and bone marrow. **A** Macroscopic picture of a purple and thick plaque at the left scapula with **B**/**C** diffuse infiltration (90%) of medium sized leukaemic blasts in respective histopathological examination of skin lesions. **D** H&E morphology at intermediate magnification of bone marrow and **E** respective staining for the myeloid-specific blast marker CD33

Admission blood cultures were positive for *Listeria monocytogenes* and *Streptococcus mitis.* Viral screening for SARS-CoV-2, HIV and Hepatitis B/C was negative. Antimicrobial regimen with piperacillin–tazobactam and caspofungin was commenced with reduction in inflammatory markers (CRP 38 to 29 mg/dl, procalcitonin 42 to 7 ng/ml, interleukin-6 2625 to 63 pg/ml). On day 4, the patient deteriorated rapidly progressing to septic shock and acute respiratory distress syndrome requiring multiorgan support. CT chest images showed dramatic progression of pulmonary changes (Fig. 2). In repeat microbiological investigations, surprisingly *Legionella pneumophila* was detected in urine and endotracheal secretions by antigen and PCR, respectively, suggestive of previously undiagnosed community-acquired Legionellosis. Due to absence of pulmonary symptoms, respiratory specimens had not been collected on admission. Legionella testing of water systems within our unit was negative. Local health protection agency was notified but investigations could not identify sources for either of two pathogens. Despite maximal treatment including escalation of antimicrobial regimen to meropenem and moxifloxacin on day 5, the patient’s condition deteriorated further [1, 2]. Life-extending measures were terminated on day 9.

**Fig. 2:**
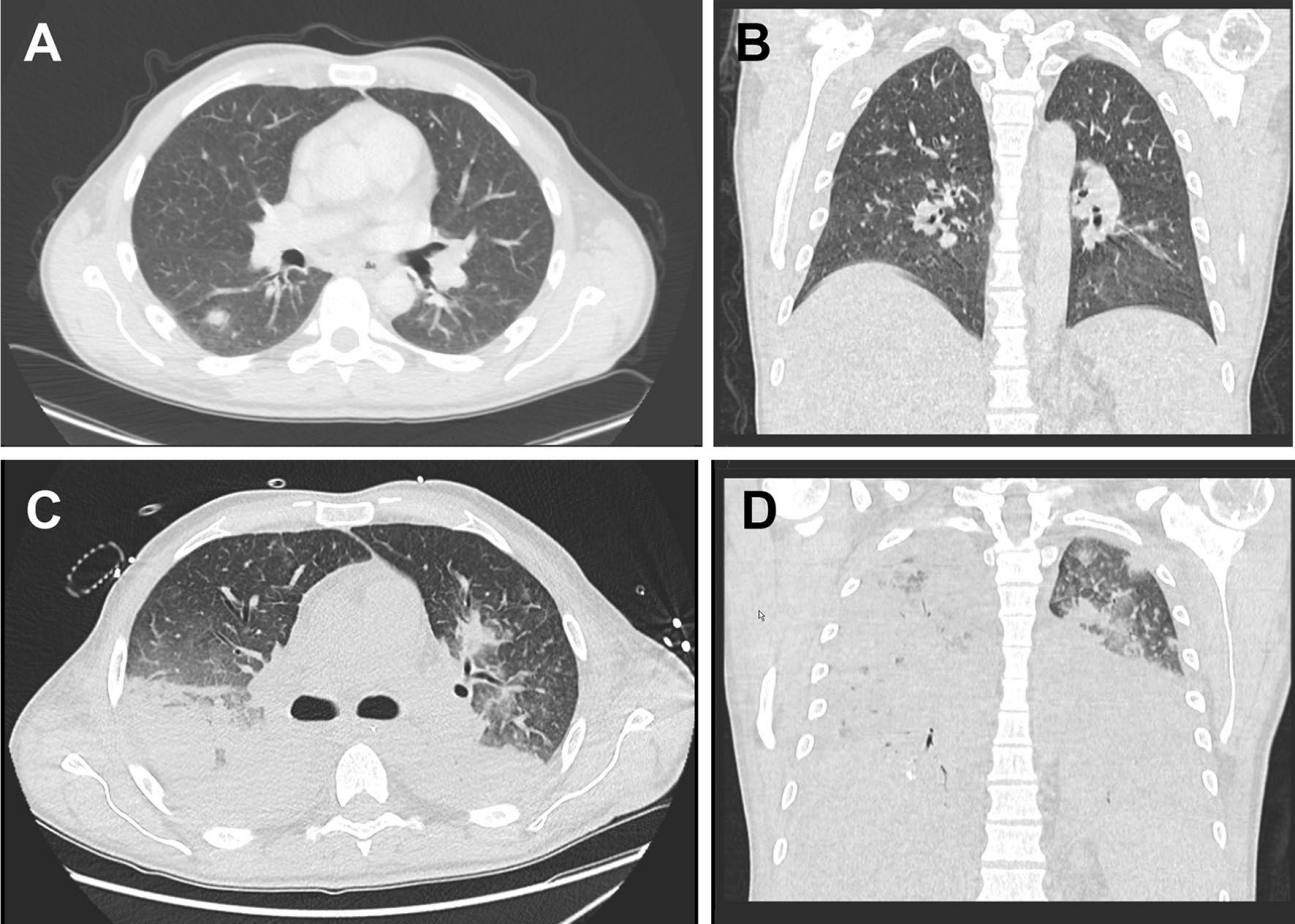
CT images show pulmonary infiltrates initially presumed to be due to fungal infection or leukaemic manifestations at the time of admission (**A**/**B**) and progressive diffuse opacities due the development of severe ARDS 5 days later (**C**/**D**)

Polymicrobial life-threatening infections are a known feared complication of hematological malignancies particularly during active immunosuppressive treatment [3–5]. However, the unique combination of community-acquired infections in this case, highlights the importance of environmental, behavioral and socio-economic risk factors as well as continued re-evaluation of differential diagnosis and therapy in managing these complex cases.

## Data Availability

Not applicable.
